# Effect of non-invasive brain stimulation on post-stroke cognitive impairment: a meta-analysis

**DOI:** 10.3389/fneur.2024.1424792

**Published:** 2024-10-16

**Authors:** Jing Zhao, Qian Meng, Shuo Qi, Hongfei Zhao, Ling Xia

**Affiliations:** Department of Rehabilitation Medicine, Zibo Central Hospital, Zibo, Shandong, China

**Keywords:** meta-analysis, post-stroke cognitive impairment, repetitive transcranial magnetic stimulation, transcranial direct current stimulation, systematic review

## Abstract

**Background:**

Previous studies have suggested that repetitive transcranial magnetic stimulation (rTMS) may be an effective and safe alternative treatment for post-stroke cognitive impairment (PSCI). Similarly, the application of transcranial direct current stimulation (tDCS) during stroke rehabilitation has been shown to improve cognitive function in PSCI patients. However, there have been conflicting results from some studies. Therefore, this study aims to conduct a meta-analysis to evaluate the effects of tDCS and rTMS on PSCI.

**Methods:**

The meta-analysis search for articles published from the initial availability date to 5 February 2024 in databases. The extracted study data were entered into STATA 12.0 software for statistical analysis.

**Results:**

This meta-analysis provides evidence that both rTMS and tDCS have a positive impact on general cognitive function in PSCI patients [immediate effect of rTMS: standard mean difference (SMD) = 2.58, 95% confidence interval (CI) = 1.44 to 3.71; long-term effect of rTMS: SMD = 2.33, 95% CI = 0.87–3.78; immediate effect of tDCS: SMD = 2.22, 95% CI = 1.31–3.12]. Specifically, rTMS was found to significantly improve attention, language, memory, and visuospatial functions, while it did not show a significant therapeutic effect on executive function (attention: SMD = 3.77, 95% CI = 2.30–5.24; executive function: SMD = −0.52, 95% CI = −3.17–2.12; language: SMD = 3.43, 95% CI = 1.50–5.36; memory: SMD = 3.52, 95% CI = 1.74–5.30; visuospatial function: SMD = 4.71, 95% CI = 2.61–6.80). On the other hand, tDCS was found to significantly improve executive and visuospatial functions but did not show a significant improvement in attention function and memory (attention: SMD = 0.63, 95% CI = −0.30–1.55; executive function: SMD = 2.15, 95% CI = 0.87–3.43; memory: SMD = 0.99, 95% CI = −0.81–2.80; visuospatial function: SMD = 2.64, 95% CI = 1.04–4.23).

**Conclusion:**

In conclusion, this meta-analysis demonstrates that both rTMS and tDCS are effective therapeutic techniques for improving cognitive function in PSCI. However, more large-scale studies are needed to further investigate the effects of these techniques on different cognitive domains in PSCI.

## 1 Introduction

Post-stroke cognitive impairment (PSCI) is a condition characterized by the impairment of cognitive functions such as attention, executive function, memory, language function, and visuospatial function within 6 months after a stroke (1). It affects a significant proportion of stroke survivors, with estimates suggesting that up to 60% may experience some degree of PSCI (2). This can have a profound impact on patients’ ability to care for themselves, participate in social activities, and maintain employment, placing a heavy burden on both their families and society as a whole (3).

Currently, the main focus of PSCI treatment is on drug therapy, specifically the use of cholinesterase inhibitors and N-methyl-D-aspartic acid receptor antagonists (4). However, research has shown that these drugs only provide short-term benefits and often come with various side effects (5). Cognitive rehabilitation training is another commonly used treatment approach aimed at promoting cognitive recovery in PSCI patients (6). However, this type of training is often hindered by poor patient cooperation, lengthy treatment times, and a lack of significant improvement (6). In recent years, two non-invasive brain stimulation (NIBS) techniques, namely repetitive transcranial magnetic stimulation (rTMS) and transcranial direct current stimulation (tDCS) have gained attention in stroke rehabilitation (7, 8). RTMS is a NIBS technique, which has garnered significant attention for its therapeutic potential in treating a spectrum of neurological and psychiatric disorders, notably depression (9). The mechanism of rTMS involves the generation of a magnetic field that permeates the skull and induces an electric current in the underlying brain tissue. This current stimulates the superficial layers of the cortex, leading to the depolarization of neuronal membranes in the targeted cortical regions (10). Given its non-invasive nature and the ability to focally modulate brain activity, rTMS is increasingly being recognized as a promising treatment modality. TDCS is another NIBS approach that modulates cortical excitability by applying a weak direct current through two scalp electrodes (11). This technique is distinguished by its practical advantages, including cost-effectiveness, ease of use, portability, and a favorable safety profile (12). Preliminary research indicates that tDCS may exert beneficial effects on certain mental health conditions, with studies suggesting its efficacy in treating major depressive disorder and schizophrenia (13). The accessibility and non-invasiveness of tDCS make it an attractive option for adjunctive therapy or as a standalone intervention in clinical settings. Previous studies have suggested that rTMS may be an effective and safe alternative treatment for PSCI (14–16). Similarly, the application of tDCS during stroke rehabilitation has been shown to improve cognitive function in PSCI patients (17, 18). However, there have been conflicting results from some studies (19–22). Therefore, this study aims to conduct a meta-analysis to evaluate the effects of NIBS (tDCS and rTMS) on PSCI.

## 2 Methods

### 2.1 Protocol

This study adhered to the Preferred Reporting Items for Systematic Reviews and Meta-Analyses (PRISMA) statement (23). As a meta-analysis, this study involved secondary analysis and did not require ethical approval.

### 2.2 Search strategy

A comprehensive search was conducted using the following databases: Web of Science, PubMed, Embase, and Google Scholar. The search included articles published from the initial availability date to 5 February 2024. The search terms used were: (“transcranial magnetic stimulation” OR “TMS” OR “transcranial direct current stimulation” OR “tDCS”) AND (“cerebrovascular” OR “stroke” OR “hemorrhage”) AND (“cognitive impairment” OR “cognitive dysfunction” OR “cognitive function”).

### 2.3 Inclusion and exclusion criteria

The inclusion criteria for the literature were as follows: (1) studies that investigated PSCI; and (2) studies that investigated rTMS or tDCS. The exclusion criteria for the literature were as follows: (1) animal studies; (2) studies that were not designed as randomized controlled trials; (3) trial groups that did not receive rTMS or tDCS treatment intervention; (4) control groups that did not receive sham rTMS or tDCS treatment or cognitive function training only; (5) studies included patients with language disorders; (6) studies that did not explore outcome indicators of cognitive function, such as the Mini-Mental State Examination (MMSE), Montreal Cognitive Assessment (MoCA), Loewenstein Occupational Therapy’s Cognitive Assessment (LOTCA), etc.; (7) studies that did not provide sufficient experimental data; and (8) reviews, meta-analyses, or case reports.

### 2.4 Data extraction

Two independent reviewers extracted data from the studies. The extracted data included the first author of the study, publication year, sample size, age of patients, gender, type of stroke, stroke location, disease duration, interventions, site of stimulation, intensity of stimulation, duration of stimulation, treatment period, outcome measure, and adverse control effect. Mean values and standard deviations (SD) of the increase or reduction rate of scores of scales for cognitive function were calculated from the included studies. These scales included MMSE, Modified Barthel Index (MBI), MoCA, LOTCA, Repeatable Battery for the Assessment of Neuropsychological Status (RBANS), Korean MMSE (K-MMSE), the Korean version of the MBI (K-MBI), Korean MoCA (K-MoCA), Korean version of the Dementia Rating Scale-2, FIM, Seoul computerized neuropsychological test (SCNT), the Tower of London test, Rivermead behavior memory test (RBMT), trail making test-A (TMT-A), digit symbol test (DST), digital span test (DS), the Wisconsin card sorting test (WCST), Stroop color-word test (SCWT), Korean-Boston naming test, Go/No Go, and controlled oral word association test, etc. In studies that used more than one scale to assess cognitive function, the mean values of the increase or reduction rate of these scores were calculated.

### 2.5 Statistical analysis

The extracted study data were entered into STATA 12.0 software for statistical analysis. The standard mean difference (SMD) and a 95% confidence interval (CI) were computed. Depending on the magnitude of heterogeneity, a random-effects model or a fixed-effects model was used for the meta-analysis. When the heterogeneity was high (*p* value for Cochran’s Q test ≤0.05 and *I*^2^ ≥ 50%), the random-effects model (DerSimonian and Laird method) was used. Conversely, the fixed-effects model (Mantel–Haenszel method) was used. Subgroup analysis was conducted to explore the source of heterogeneity, considering different ethnicities, different sites of stimulation, and different frequencies of rTMS. A meta-regression was performed to examine whether publication year, age of patients, gender, disease duration, intensity of stimulation, duration of stimulation, and treatment period moderated the effects of rTMS or tDCS on PSCI. Sensitivity analyses were conducted to assess the robustness of the findings. Publication bias was assessed using the funnel plot, Egger’s test, and Begg’s test.

## 3 Results

### 3.1 Study selection and study characteristics

The results of the search are summarized in Supplementary Figure 1. A total of 912 unique records were identified from the initial availability date to 5 February 2024. After an initial screening of titles and abstracts, 425 studies that did not investigate rTMS or tDCS were eliminated. Subsequently, 216 studies not exploring PSCI were excluded, along with 115 animal studies. Furthermore, 32 studies lacking a randomized controlled trial design were removed, as were 26 reviews, meta-analyses, and case reports. This process resulted in 98 studies for full-text review. Upon thorough examination, 22 studies without trial groups receiving rTMS or tDCS treatment were excluded, as were 15 studies with control groups lacking sham rTMS or tDCS treatment or those receiving only cognitive function training. Additionally, 22 studies that did not assess cognitive function outcomes and 16 studies deficient in experimental data were excluded. Ultimately, 23 studies were selected for the final review (14–22, 24–37). The main characteristics of the included studies are presented in Supplementary Tables 1, 2. Among the finally included 23 studies, 13 studies (14–16, 21, 22, 24, 31–37) involving 275 PSCI patients in the experimental group and 257 PSCI patients in the control group, investigating the effects of rTMS on PSCI. Additionally, 11 studies (17–20, 24–30) including 230 PSCI patients in the experimental group and 205 PSCI patients in the control group explored the effects of tDCS on PSCI.

### 3.2 Results of statistical analysis

#### 3.2.1 Effect of rTMS on general cognitive function in PSCI

**Figure 1:**
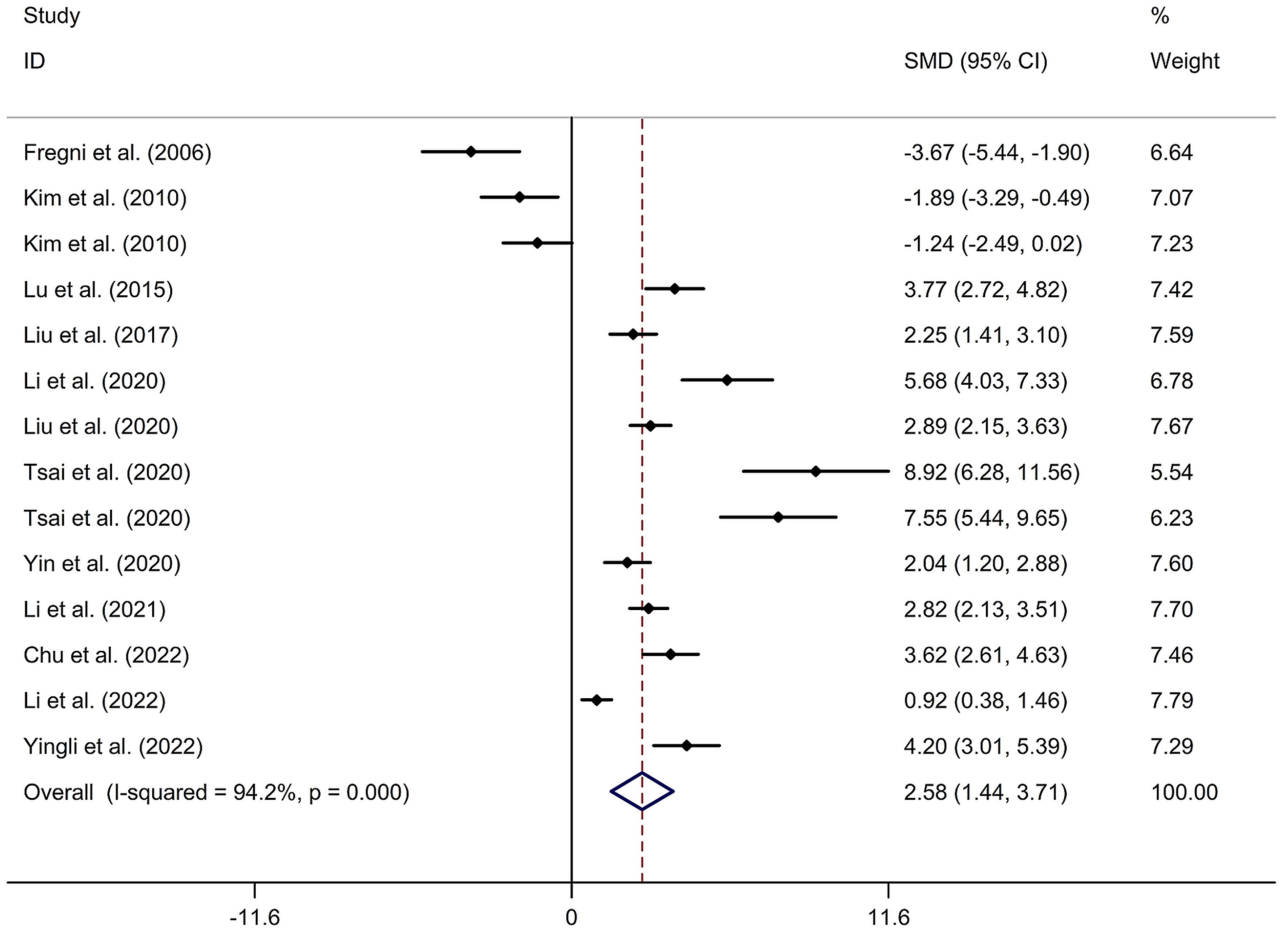
Forest plot regarding the immediate effect of rTMS on general cognitive function in PSCI. CI, confidence interval; PSCI, post-stroke cognitive impairment; rTMS, repetitive transcranial magnetic stimulation; SMD, standard mean difference.

**Figure 2:**
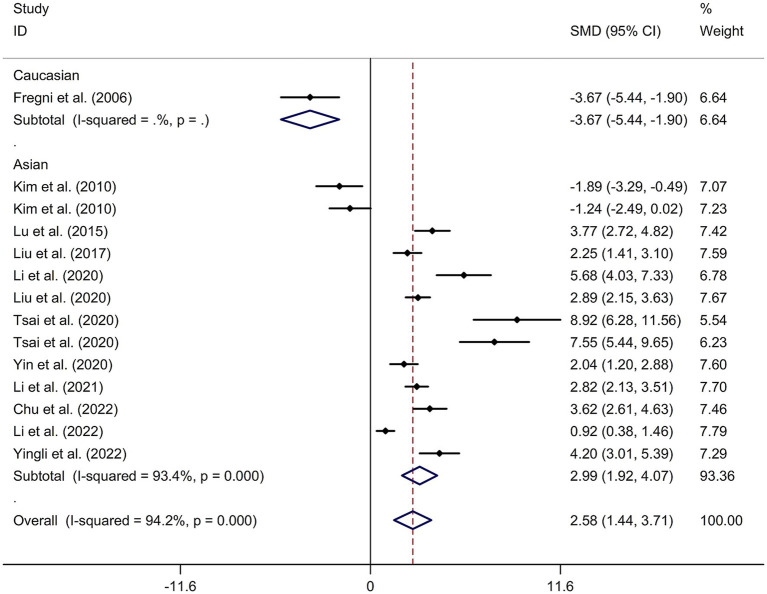
Subgroup analysis regarding the immediate effect of rTMS on general cognitive function in PSCI with different races. CI, confidence interval; PSCI, post-stroke cognitive impairment; rTMS, repetitive transcranial magnetic stimulation; SMD, standard mean difference.

**Figure 3:**
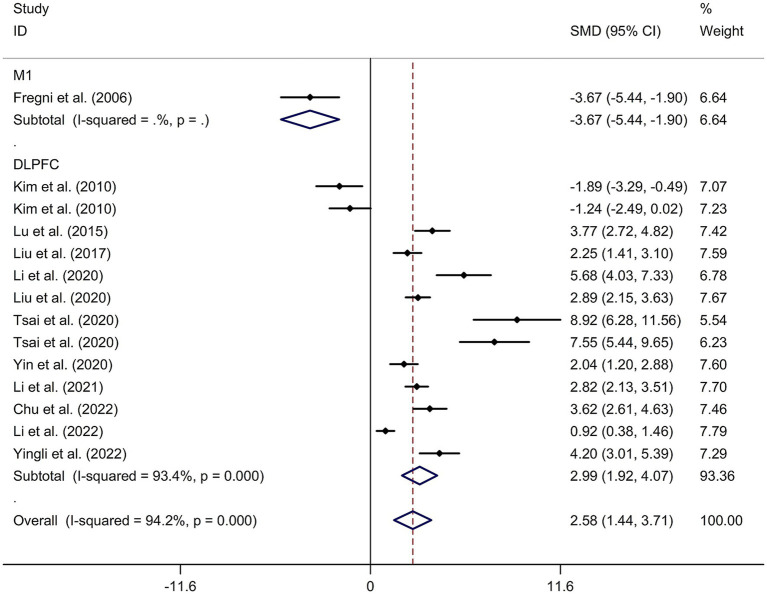
Subgroup analysis regarding the immediate effect of rTMS with different stimulation sites on general cognitive function in PSCI. CI, confidence interval; PSCI, post-stroke cognitive impairment; rTMS, repetitive transcranial magnetic stimulation; SMD, standard mean difference.

**Figure 4:**
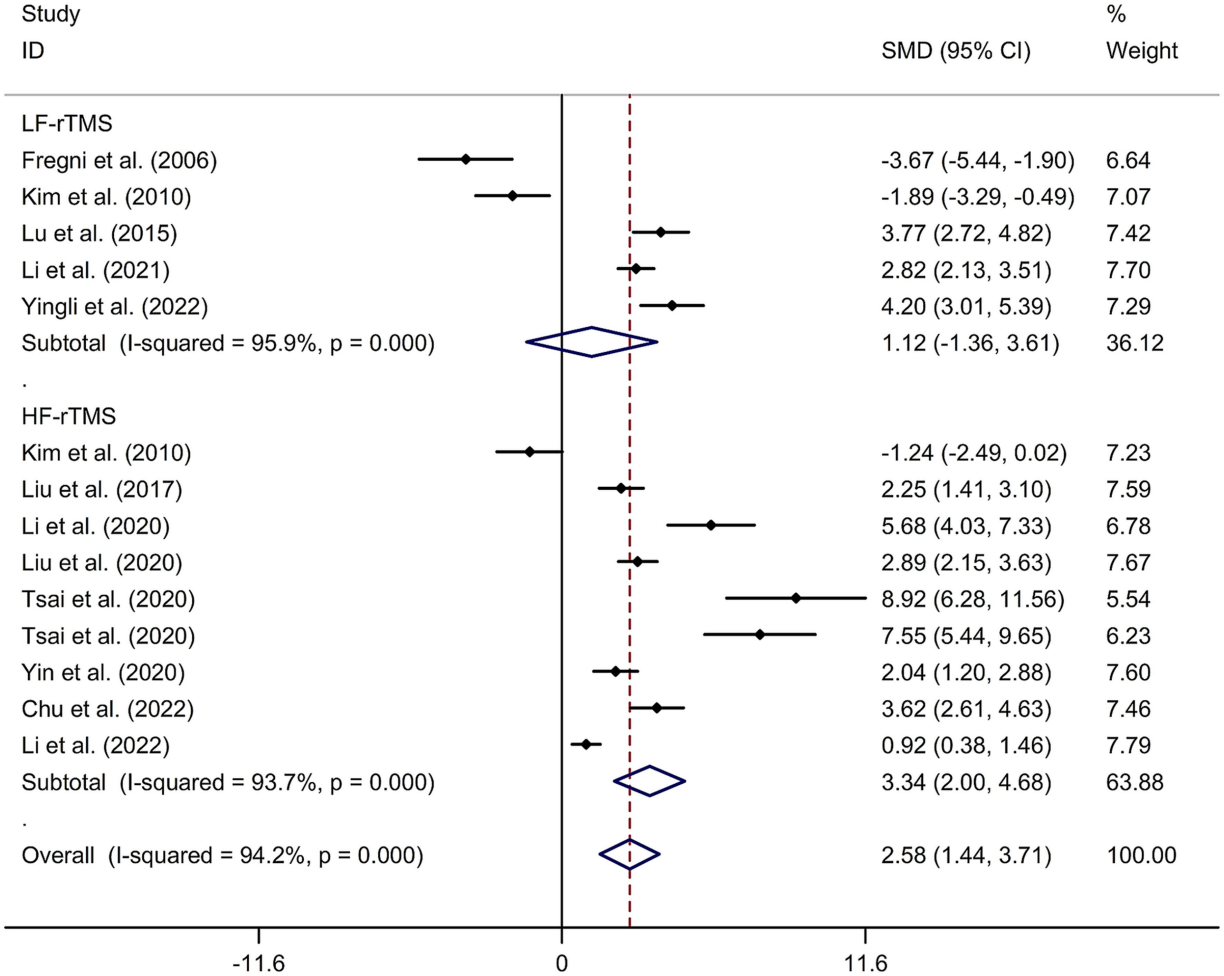
Subgroup analysis regarding the immediate effect of rTMS with different frequencies on general cognitive function in PSCI. CI, confidence interval; PSCI, post-stroke cognitive impairment; rTMS, repetitive transcranial magnetic stimulation; SMD, standard mean difference.

A total of 12 randomized controlled trials (14–16, 21, 22, 24, 32–37) were included in this meta-analysis to investigate the immediate effect of rTMS on general cognitive function in PSCI. The analysis included 245 patients treated with rTMS and 290 patients in the control groups. Due to significant heterogeneity among the studies (*I*^2^ = 94.2%, *p* value for Cochran’s Q test <0.001; Figure 1), a random-effects model was used. The results showed that PSCI patients in the rTMS group had a significantly greater improvement in general cognitive function immediately after treatment compared to those in the sham rTMS or no rTMS intervention group (SMD = 2.58, 95% CI = 1.44–3.71; Figure 1). Subgroup analysis was conducted to explore the effect of rTMS on general cognitive function in specific populations. The analysis showed that Asian PSCI patients who received rTMS immediately after treatment had a greater improvement in general cognitive function compared to those who received sham tDCS or no rTMS intervention (SMD = 2.99, 95% CI = 1.92–4.07; Figure 2). *N* = 11 studies used DLPFC as stimulation site. PSCI patients who received rTMS on the DLPFC had a greater improvement in general cognitive function compared to those who received sham rTMS or no rTMS intervention (SMD = 2.99, 95% CI = 1.92–4.07; Figure 3). Only one study used primary motor cortex (M1) as stimulation site. Further subgroup analysis based on the frequency of rTMS stimulation revealed that PSCI patients who received high-frequency (HF) rTMS had a greater improvement in general cognitive function compared to those who received sham rTMS or no rTMS intervention. However, no significant difference in the change of general cognitive function was observed between patients who received low-frequency rTMS and those who received sham rTMS or no rTMS intervention (HF-rTMS: SMD = 3.34, 95% CI = 2.00–4.68; LF-rTMS: SMD = 1.12, 95% CI = −1.36–3.61; Figure 4). Meta-regression analysis was conducted to explore potential factors contributing to heterogeneity in the effect of rTMS on general cognitive function in PSCI. The analysis revealed that variables such as age of patients, gender, disease duration, intensity of stimulation, duration of stimulation, and treatment period did not significantly impact the heterogeneity. The only significant moderator was the publication year (*p* = 0.005). Sensitivity analysis indicated that no single study had a significant impact on the pooled effect size (Supplementary Figure 2). The funnel plot (Supplementary Figure 3) showed no evidence of publication bias, which was further supported by the results of Egger’s test and Begg’s test (Egger’s test: *p* = 0.434; Begg’s test: *p* = 0.189).

**Figure 5:**
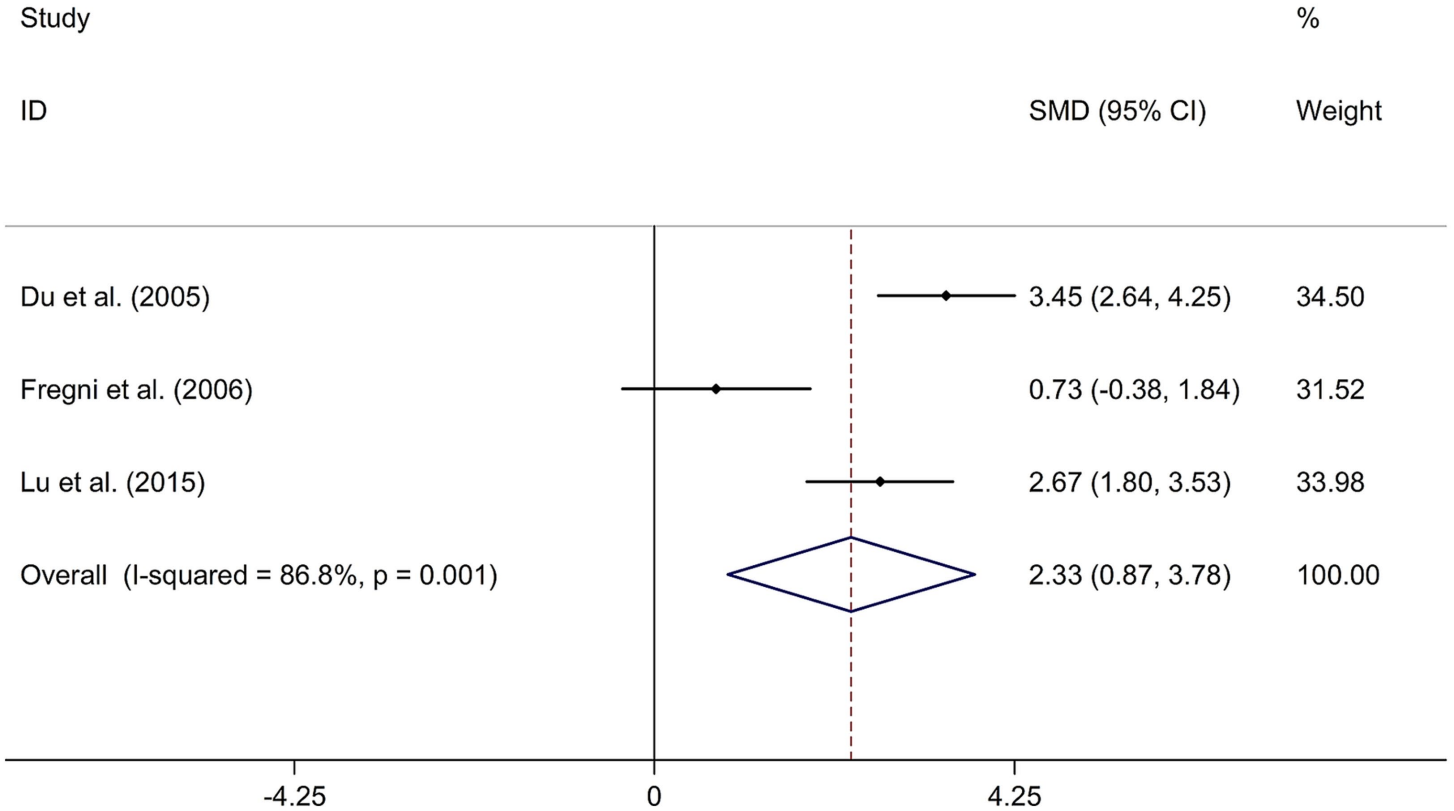
Forest plot regarding the long-term effect of rTMS on general cognitive function in PSCI. CI, confidence interval; PSCI, post-stroke cognitive impairment; rTMS, repetitive transcranial magnetic stimulation; SMD, standard mean difference.

To investigate the long-term effect of rTMS on general cognitive function in PSCI, three randomized controlled trials (14, 21, 31) involving 59 patients treated with rTMS and 56 patients in the control groups were included. The random-effects model was used due to significant heterogeneity among the studies (*I*^2^ = 86.8%, *p* value for Cochran’s Q test = 0.001; Figure 5). The results showed that PSCI patients in the rTMS group had a significantly greater improvement in general cognitive function in the days following treatment compared to those in the sham rTMS or no rTMS intervention group (SMD = 2.33, 95% CI = 0.87–3.78; Figure 5). Two studies used DLPFC as stimulation site. One study used M1 as stimulation site. The sensitivity analysis indicated that no single study impacted the pooled effect size (Supplementary Figure 4). Funnel plot, Egger’s test and Begg’s test showed no significant risk of publication bias (Supplementary Figure 5; Egger’s test: *p* = 0.080; Begg’s test: *p* = 0.296).

#### 3.2.2 Effect of rTMS on attention function in PSCI

To explore the immediate effect of rTMS on attention function in PSCI, six RCTs (22, 24, 32, 34–36) involving 116 patients treated with rTMS and 100 in the control groups were included. Due to significant heterogeneity among the studies (*I*^2^ = 93.8%, *p* value for Cochran’s Q test <0.001; Supplementary Figure 6), a random-effects model was used. The results showed that PSCI patients in the rTMS group had a greater improvement in attention function immediately after treatment compared to those in the sham rTMS or no rTMS intervention group (SMD = 3.77, 95% CI = 2.30–5.24; Supplementary Figure 6). All included studies used DLPFC as stimulation site. Subgroup analysis based on the frequency of rTMS stimulation revealed that PSCI patients who received HF-rTMS had a greater improvement in attention function compared to those who received sham rTMS or no rTMS intervention (SMD = 3.88, 95% CI = 1.90–5.86). Only two studies used LF-rTMS. Meta-regression analysis revealed that variables such as publication year, age of patients, gender, and disease duration did not significantly impact the heterogeneity in the effect of rTMS on attention function in PSCI, whereas intensity of stimulation, duration of stimulation, and treatment period significantly impacted the heterogeneity (intensity of stimulation: *p* = 0.014; duration of stimulation: *p* = 0.002; treatment period: *p* = 0.004). Sensitivity analysis indicated that no single study had a significant impact on the pooled effect size. Egger’s test and Begg’s test showed a significant publication bias (Egger’s test: *p* = 0.023; Begg’s test: *p* = 0.043).

#### 3.2.3 Effect of rTMS on executive function in PSCI

For executive function, three RCTs (22, 33, 36) involving 56 patients treated with rTMS and 54 in the control groups were included. The study found no significant difference in the change of executive function between PSCI patients given rTMS and those given sham rTMS or no rTMS intervention using a random-effects model (SMD = −0.52, 95% CI = −3.17–2.12; *I*^2^ = 95.1%, *p* value for Cochran’s *Q* test <0.001; Supplementary Figure 7). All included studies used DLPFC as stimulation site. Subgroup analysis based on the frequency of rTMS stimulation revealed no significant difference in the change of executive function between PSCI patients given HF-rTMS and those given sham rTMS or no rTMS intervention (SMD = 0.80, 95% CI = −1.79–3.38). Only one study used LF-rTMS. Meta-regression analysis revealed that variables such as publication year, age of patients, gender, disease duration, intensity of stimulation, duration of stimulation, and treatment period did not significantly impact the heterogeneity in the effect of rTMS on executive function in PSCI. Sensitivity analysis indicated that no single study had a significant impact on the pooled effect size. Egger’s test, Begg’s test and funnel plot showed no significant publication bias (Egger’s test: *p* = 0.617; Begg’s test: *p* = 0.308).

#### 3.2.4 Effect of rTMS on language function in PSCI

To examine the immediate effect of rTMS on language function in PSCI, five RCTs (14, 32, 33, 35, 36) involving 122 patients treated with rTMS and 116 in the control groups were included. The study showed a greater improvement in language function in PSCI immediately after rTMS treatment compared to sham rTMS or no rTMS intervention using a random-effects model (SMD = 3.43, 95% CI = 1.50–5.36; *I*^2^ = 95.2%, *p* value for Cochran’s Q test <0.001; Supplementary Figure 8). All included studies used DLPFC as stimulation site. Subgroup analysis based on the frequency of rTMS stimulation revealed that PSCI patients who received HF-rTMS had a greater improvement in language function compared to those who received sham rTMS or no rTMS intervention (SMD = 4.76, 95% CI = 1.22–8.31). Only one study used LF-rTMS. Meta-regression analysis revealed that variables such as publication year, age of patients, gender, disease duration, intensity of stimulation, duration of stimulation, and treatment period did not significantly impact the heterogeneity in the effect of rTMS on language function in PSCI. Sensitivity analysis indicated that no single study had a significant impact on the pooled effect size. Egger’s test, Begg’s test and funnel plot showed no significant publication bias (Egger’s test: *p* = 0.081; Begg’s test: *p* = 0.086).

#### 3.2.5 Effect of rTMS on memory in PSCI

Similarly, for memory function, five RCTs (14, 32, 33, 35, 36) involving 122 patients treated with rTMS and 116 in the control groups were included. The study demonstrated a greater improvement in memory in PSCI immediately after rTMS treatment compared to sham rTMS or no rTMS intervention using a random-effects model (SMD = 3.52, 95% CI = 1.74–5.30; *I*^2^ = 95.8%, *p* value for Cochran’s Q test <0.001; Supplementary Figure 9). All included studies used DLPFC as stimulation site. Subgroup analysis based on the frequency of rTMS stimulation revealed that PSCI patients who received HF-rTMS had a greater improvement in memory compared to those who received sham rTMS or no rTMS intervention (SMD = 3.76, 95% CI = 1.07–6.45). Only two studies used LF-rTMS. Meta-regression analysis revealed that variables such as publication year, age of patients, gender, and disease duration did not significantly impact the heterogeneity in the effect of rTMS on memory in PSCI, whereas intensity of stimulation, duration of stimulation, and treatment period significantly impacted the heterogeneity (intensity of stimulation: *p* = 0.012; duration of stimulation: *p* = 0.010; treatment period: *p* = 0.013). Sensitivity analysis indicated that no single study had a significant impact on the pooled effect size. Egger’s test, Begg’s test and funnel plot showed a significant publication bias (Egger’s test: *p* = 0.014; Begg’s test: *p* = 0.039).

#### 3.2.6 Effect of rTMS on visuospatial function in PSCI

Lastly, to assess the immediate effect of rTMS on visuospatial function in PSCI, four RCTs (24, 32, 33, 35) involving 108 patients treated with rTMS and 97 in the control groups were included. The study revealed a greater improvement in visuospatial function in PSCI immediately after rTMS treatment compared to sham rTMS or no rTMS intervention using a random-effects model (SMD = 4.71, 95% CI = 2.61–6.80; *I*^2^ = 95.9%, *p* value for Cochran’s Q test <0.001; Supplementary Figure 10). All included studies used DLPFC as stimulation site. Subgroup analysis based on the frequency of rTMS stimulation revealed that PSCI patients who received HF-rTMS had a greater improvement in visuospatial function compared to those who received sham rTMS or no rTMS intervention (SMD = 6.18, 95% CI = 2.49–9.87). Only one study used LF-rTMS. Meta-regression analysis revealed that variables such as publication year, age of patients, gender, disease duration, intensity of stimulation, duration of stimulation, and treatment period did not significantly impact the heterogeneity in the effect of rTMS on visuospatial function in PSCI. Sensitivity analysis indicated that no single study had a significant impact on the pooled effect size. Egger’s test, Begg’s test and funnel plot showed a significant publication bias (Egger’s test: *p* = 0.004; Begg’s test: *p* = 0.049).

#### 3.2.7 Effect of tDCS on general cognitive function in PSCI

**Figure 6:**
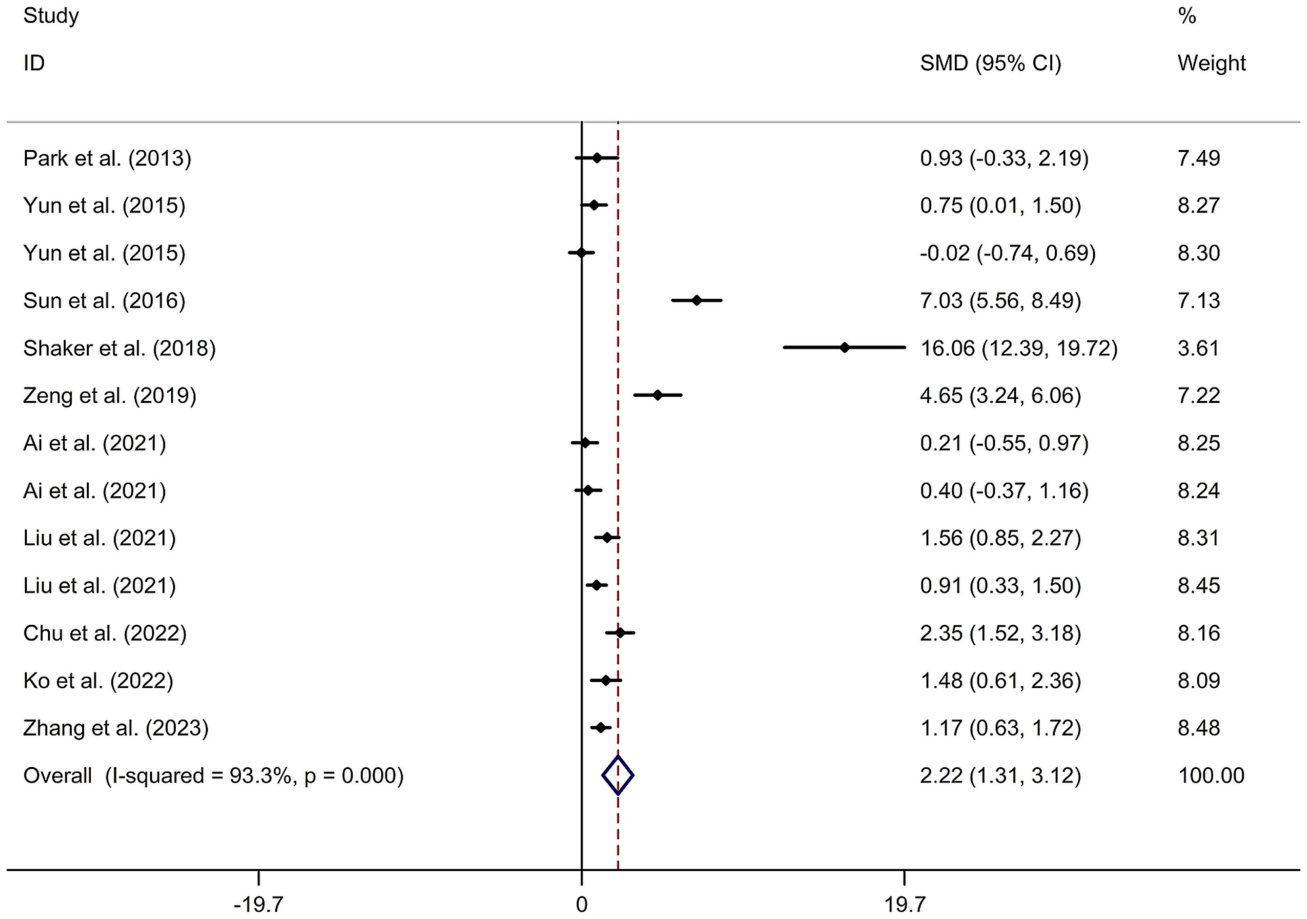
Forest plot regarding the immediate effect of tDCS on general cognitive function in PSCI. CI, confidence interval; PSCI, post-stroke cognitive impairment; SMD, standard mean difference; tDCS, transcranial direct current stimulation.

**Figure 7:**
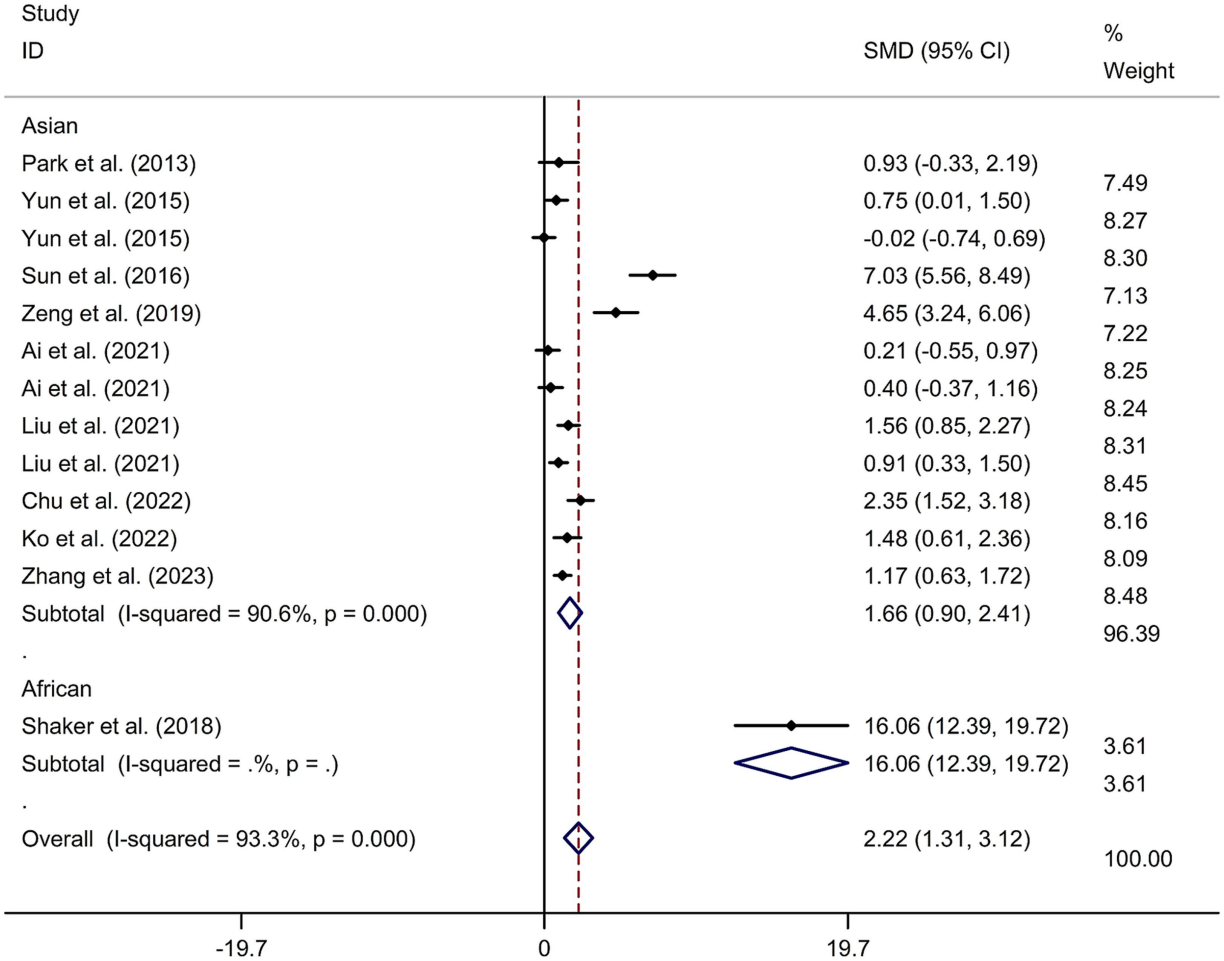
Forest plot regarding the immediate effect of tDCS on general cognitive function in PSCI with different races. CI, confidence interval; PSCI, post-stroke cognitive impairment; SMD, standard mean difference; tDCS, transcranial direct current stimulation.

**Figure 8:**
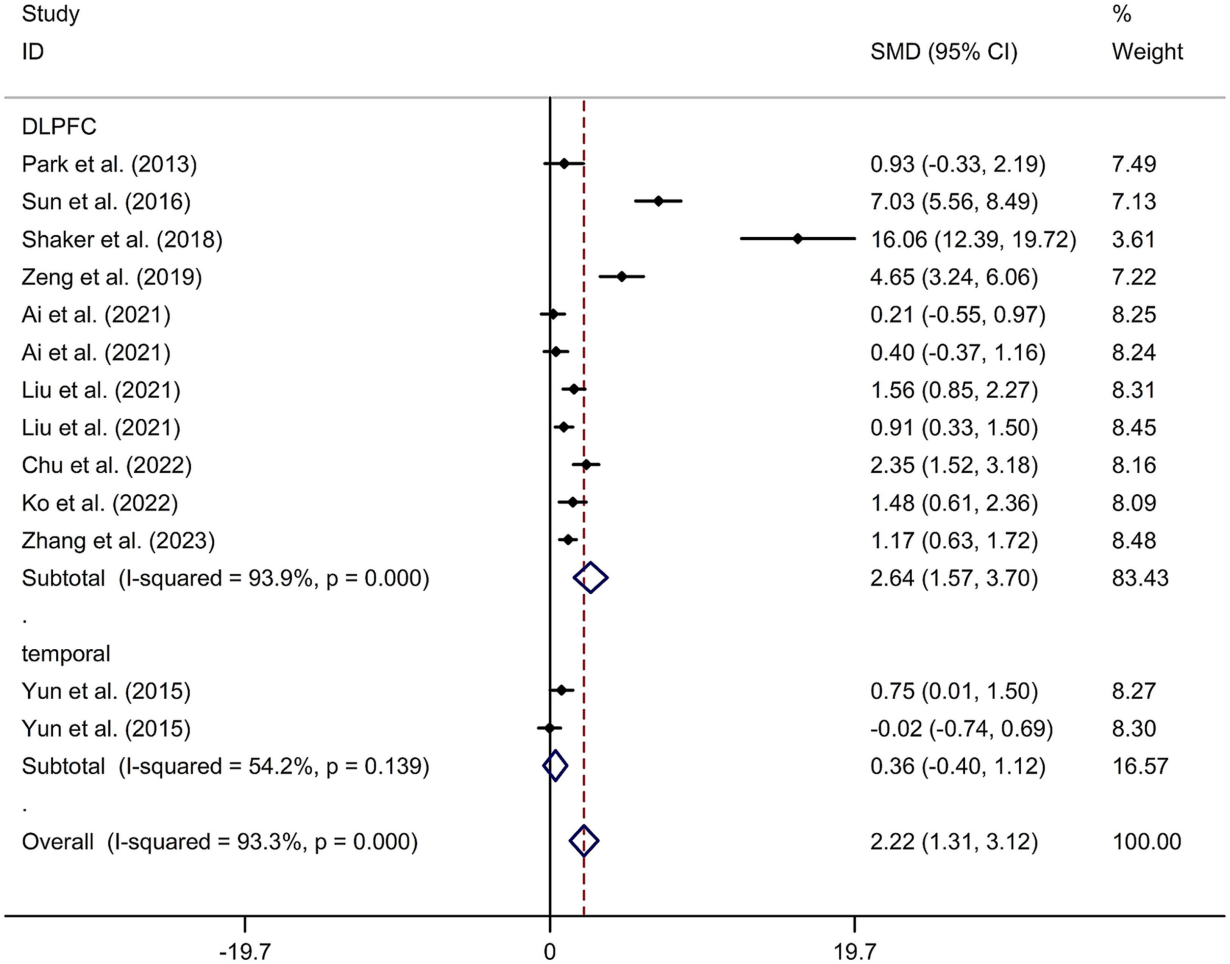
Forest plot regarding the immediate effect of tDCS with different stimulation sites on general cognitive function in PSCI. CI, confidence interval; PSCI, post-stroke cognitive impairment; SMD, standard mean difference; tDCS, transcranial direct current stimulation.

This study included 11 RCTs (17–20, 24–30) involving a total of 231 patients treated with tDCS and 204 patients in the control groups. The random-effects model was used due to significant heterogeneity among the studies (*I*^2^ = 93.3%, *p* value for Cochran’s Q test <0.001; Figure 6). The findings revealed that PSCI patients in the tDCS group experienced a greater improvement in general cognitive function immediately after treatment, compared to those in the sham tDCS or no tDCS intervention group (SMD = 2.22, 95% CI = 1.31–3.12; Figure 6). Subgroup analysis further demonstrated that Asian PSCI patients who received tDCS treatment had a significantly greater improvement in general cognitive function compared to those who received sham tDCS or no tDCS intervention (SMD = 1.66, 95% CI = 0.90–2.41; Figure 7). Additionally, subgroup analysis indicated that PSCI patients who received tDCS on the DLPFC showed a greater improvement in general cognitive function compared to those who received sham tDCS or no tDCS intervention (SMD = 2.64, 95% CI = 1.57–3.70; Figure 8). Only one study used temporal lobe as stimulation site. Meta-regression analysis revealed that the examined variables, including publication year, age of patients, gender, disease duration, intensity of stimulation, duration of stimulation, and treatment period, did not significantly impact the heterogeneity in the effect of tDCS on general cognitive function in PSCI. Sensitivity analysis confirmed that no single study had a substantial impact on the overall effect size (Supplementary Figure 11). However, the funnel plot, Egger’s test, and Begg’s test indicated a significant risk of publication bias (Supplementary Figure 12; Egger’s test: *p* = 0.002; Begg’s test: *p* = 0.033).

#### 3.2.8 Effect of tDCS on attention function in PSCI

Eleven RCTs (17–20, 24–30) involving 231 patients treated with tDCS and 204 in the control groups were included to investigate the immediate effect of tDCS on attention function in PSCI. The study findings did not show a significant difference in the change of attention function between PSCI patients who received tDCS and those who received sham tDCS or no tDCS intervention, as determined by a random-effects model (SMD = 0.63, 95% CI = −0.30–1.55; *I*^2^ = 94.6%, *p* value for Cochran’s Q test <0.001; Supplementary Figure 13). Subgroup analysis did not show a significant difference in the change of attention function between PSCI patients who received tDCS on the DLPFC and those who received sham tDCS or no tDCS intervention (SMD = 0.90, 95% CI = −0.15–1.95). Only one study used temporal lobe as stimulation site. Meta-regression analysis revealed that the examined variables, including publication year, age of patients, gender, disease duration, intensity of stimulation, duration of stimulation, and treatment period, did not significantly impact the heterogeneity in the effect of tDCS on attention function in PSCI. Sensitivity analysis confirmed that no single study had a substantial impact on the overall effect size. The funnel plot, Egger’s test, and Begg’s test indicated no significant risk of publication bias (Egger’s test: *p* = 0.135; Begg’s test: *p* = 0.200).

#### 3.2.9 Effect of tDCS on executive function in PSCI

Eight RCTs (17–20, 25, 26, 29, 30) involving 166 patients treated with tDCS and 137 in the control groups were included to examine the immediate effect of tDCS on executive function in PSCI. The study revealed a greater improvement in executive function in PSCI immediately after tDCS treatment, compared to sham tDCS or no tDCS intervention, as determined by a random-effects model (SMD = 2.15, 95% CI = 0.87–3.43; *I*^2^ = 93.8%, *p* value for Cochran’s Q test <0.001; Supplementary Figure 14). Subgroup analysis indicated that PSCI patients who received tDCS on the DLPFC showed a greater improvement in executive function compared to those who received sham tDCS or no tDCS intervention (SMD = 1.63, 95% CI = 0.27–2.98). Only one study used temporal lobe as stimulation site. Meta-regression analysis revealed that the examined variables, including publication year, age of patients, gender, disease duration, intensity of stimulation, duration of stimulation, and treatment period, did not significantly impact the heterogeneity in the effect of tDCS on executive function in PSCI. Sensitivity analysis confirmed that no single study had a substantial impact on the overall effect size. The funnel plot, Egger’s test, and Begg’s test indicated no significant risk of publication bias (Egger’s test: *p* = 0.122; Begg’s test: *p* = 0.076).

#### 3.2.10 Effect of tDCS on memory in PSCI

Three RCTs (18, 27, 29) involving a total of 80 patients treated with tDCS and 65 patients in the control groups were included to investigate the immediate effect of tDCS on memory in PSCI. The analysis showed no significant difference in the change of memory between PSCI patients who received tDCS and those who received sham tDCS or no tDCS intervention, as determined by a random-effects model (SMD = 0.99, 95% CI = −0.81–2.80; *I*^2^ = 95.8%, *p* value for Cochran’s Q test <0.001; Supplementary Figure 15). Two studies used DLPFC as stimulation site. Only one study used temporal lobe as stimulation site. Sensitivity analysis confirmed that no single study had a substantial impact on the overall effect size. The funnel plot, Egger’s test, and Begg’s test indicated a significant risk of publication bias (Egger’s test: *p* = 0.014; Begg’s test: *p* = 0.042).

#### 3.2.11 Effect of tDCS on visuospatial function in PSCI

Three RCTs (18, 24, 30) involving a total of 64 patients treated with tDCS and 65 patients in the control groups were included to explore the immediate effect of tDCS on visuospatial function in PSCI. The analysis revealed a significant improvement in visuospatial function in PSCI immediately after tDCS treatment, compared with sham tDCS or no tDCS intervention, as determined by a random-effects model (SMD = 2.64, 95% CI = 1.04–4.23; *I*^2^ = 89.9%, *p* value for Cochran’s Q test <0.001; Supplementary Figure 16). All these studies used DLPFC as stimulation site. Sensitivity analysis confirmed that no single study had a substantial impact on the overall effect size. The funnel plot, Egger’s test, and Begg’s test indicated no significant risk of publication bias (Egger’s test: *p* = 0.154; Begg’s test: *p* = 0.296).

## 4 Discussion

This meta-analysis provides evidence that both rTMS and tDCS have a positive impact on general cognitive function in PSCI patients. Specifically, rTMS was found to significantly improve attention, language, memory, and visuospatial functions, while it did not show a significant therapeutic effect on executive function. On the other hand, tDCS was found to significantly improve executive and visuospatial functions but did not show a significant improvement in attention function and memory.

The etiology of PSCI remains an enigmatic area of research, with its pathophysiological underpinnings not yet fully elucidated. The onset of PSCI could be attributed to the cerebral infarction itself, or it might stem from the exacerbation of underlying vascular risk factors in the wake of a stroke event, such as pre-existing white matter pathology or concomitant neurodegenerative processes (38). Additionally, a reduction in the concentration of key neurotransmitters, notably acetylcholine and dopamine, has been observed in individuals with PSCI (39). Recent studies have shed light on the potential therapeutic mechanisms of non-invasive brain stimulation techniques in PSCI. Chen et al. demonstrated that HF-rTMS can enhance cognitive performance and reduce white matter damage in a rat model of PSCI. This effect was associated with a phenotypic shift in microglia toward the anti-inflammatory M2 phenotype (40). Furthermore, TMS has been shown to elevate extracellular dopamine and glutamate levels within the brain, suggesting its potential in modulating neurotransmitter dynamics (41). TDCS is another promising NIBS approach that modulates cortical excitability. Anodal tDCS is hypothesized to enhance excitatory synaptic transmission by promoting glutamatergic signaling and concurrently inhibiting gamma-aminobutyric acid (GABAergic) neurotransmission in the cortex. This results in a fine-tuned adjustment of the cortical excitation-inhibition balance. Moreover, tDCS has been reported to modulate the activity of various neurotransmitter systems, including dopamine, serotonin, and acetylcholine, either positively or negatively, depending on the stimulation parameters and the specific brain region targeted (42). The cumulative effects of multi-session tDCS are believed to augment the efficiency of information processing within the cerebral cortex by inducing long-term potentiation (LTP), a process that involves the synthesis of various proteins crucial for synaptic plasticity and memory consolidation (42). These findings underscore the potential of NIBS techniques in rebalancing neural networks and enhancing cognitive function in PSCI, offering a viable avenue for future therapeutic development.

The therapeutic effect of rTMS on PSCI is believed to be influenced by factors such as stimulation site and rTMS parameters (22). Therefore, selecting the appropriate treatment mode and parameters is crucial for successful therapeutic applications of rTMS. Most of the studies included in this meta-analysis used stimulation of the DLPFC. This is likely because stimulation of the DLPFC can restore the impaired cholinergic innervation from the basal forebrain, which is typically seen in patients with mild cognitive impairment (MCI) (43), and is important for memory tasks in healthy individuals (44). The DLPFC is considered to be a key node in brain networks involved in working memory and executive control (45). By stimulating this area, it is possible to regulate the function of this brain network and promote cognitive recovery in patients.

The meta-analysis also revealed that HF-rTMS showed significant therapeutic effects (46), while LF-rTMS did not. HF-rTMS works by facilitating the excitation of the targeted area, thus promoting post-stroke recovery (47). On the other hand, LF-rTMS works by inhibiting excitability in the contra-lesional hemisphere to the lesioned hemisphere, restoring balance and enabling the undamaged parts of the functional area to function properly (47). These findings are consistent with a recent meta-analysis (48) that reported improved overall cognitive function in PSCI patients with HF-rTMS compared to non-rTMS or sham rTMS. However, it is important to note that only three studies explored the effect of rTMS on executive function in PSCI, highlighting the need for further research in this area.

The meta-analysis findings suggest that tDCS has a positive therapeutic effect on cognitive function in patients with PSCI. Specifically, anodal tDCS was found to improve general cognitive performance compared to passive tDCS, although no significant difference was observed in memory performance between the two groups (49). Previous research has indicated that tDCS can modulate local cortical excitability, leading to improvements in interhemispheric inhibition (50, 51). Additionally, tDCS has been shown to influence regional cerebral blood flow and mitigate aberrant neural synchronization (52). However, it is important to note that only three studies have investigated the therapeutic effect of tDCS on memory and visuospatial function in PSCI. Therefore, further research is needed to explore the impact of tDCS on various cognitive domains in PSCI.

A variety of non-invasive brain stimulation (NIBS) techniques have demonstrated efficacy in the context of stroke rehabilitation, offering diverse mechanisms of action and potential benefits for post-stroke cognitive impairment (PSCI). Among these are transcranial electrical stimulation (tES), transcranial focused ultrasound stimulation (tFUS), and transcutaneous vagus nerve stimulation (tVNS), each of which has been shown to contribute positively to the recovery process (53). TES, which includes transcranial direct current stimulation (tDCS) and transcranial alternating current stimulation (tACS), applies sinusoidal or biphasic currents to the cortex. This method modulates endogenous brain oscillations and enhances synaptic plasticity, leading to improvements in long-term brain function and alleviation of depressive symptoms, which are common sequelae of stroke (54). TFUS is an innovative NIBS modality that has gained traction due to its precise spatial resolution and its ability to target deeper brain structures (55). The mechanism of tFUS involves the generation of nanobubbles in the neuronal cell membrane (56), which increases membrane permeability and facilitates action potential propagation. This results in enhanced interneuronal synaptic transmission, selectively activating local brain regions and modulating the excitability of neural circuits (57). TVNS, a technique that stimulates the auricular branch of the vagus nerve through the skin, holds promise for enhancing central noradrenergic activity, which is implicated in the regulation of arousal and attention. By exerting neuromodulatory effects, tVNS not only transmits electrical stimulation to the brain but also promotes neural plasticity, potentially leading to improved clinical outcomes in stroke patients (58). The therapeutic potential of these NIBS techniques lies in their ability to modulate the neurophysiological underpinnings of PSCI, offering alternative or complementary approaches to conventional rehabilitation strategies.

There are some limitations to consider in this study. Firstly, there were not enough studies available to examine the therapeutic effect of tDCS on language function in PSCI. Additionally, important information such as the type and severity of stroke, smoking and alcohol consumption, and other factors that may influence the results were not obtained from the original articles.

## 5 Conclusion

In conclusion, this meta-analysis demonstrates that both rTMS and tDCS are effective therapeutic techniques for improving cognitive function in PSCI. As such, they warrant further investigation to determine their efficacy and optimal application in the treatment of PSCI and other neurological conditions. However, more large-scale studies are needed to further investigate the effects of these techniques on different cognitive domains in PSCI.

## Data Availability

The original contributions presented in the study are included in the article/Supplementary material, further inquiries can be directed to the corresponding author.
