# Supplementary material for: Effect of non-invasive brain stimulation on post-stroke cognitive impairment: a meta-analysis
Source: Front Neurol. 2024 Oct 16;15:1424792. doi: 10.3389/fneur.2024.1424792 (PMC11521814; doi:10.3389/fneur.2024.1424792)
Supplement: Supplementary file 3 [file Image_3.pdf]

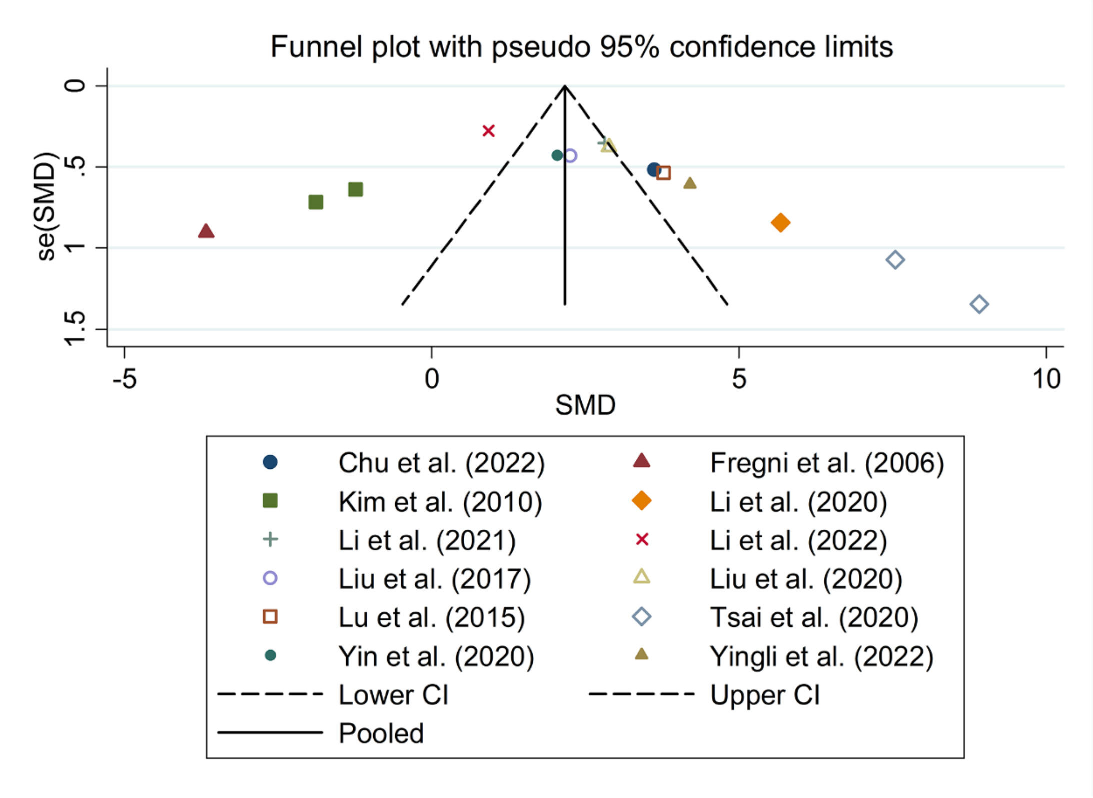

Supplementary Figure 3. Funnel plot regarding the immediate effect of rTMS on general cognitive function in PSCI. Abbreviations: PSCI, post-stroke cognitive impairment; rTMS, repetitive transcranial magnetic stimulation.
