# Supplementary material for: Effect of non-invasive brain stimulation on post-stroke cognitive impairment: a meta-analysis
Source: Front Neurol. 2024 Oct 16;15:1424792. doi: 10.3389/fneur.2024.1424792 (PMC11521814; doi:10.3389/fneur.2024.1424792)
Supplement: Supplementary file 8 [file Image_8.pdf]

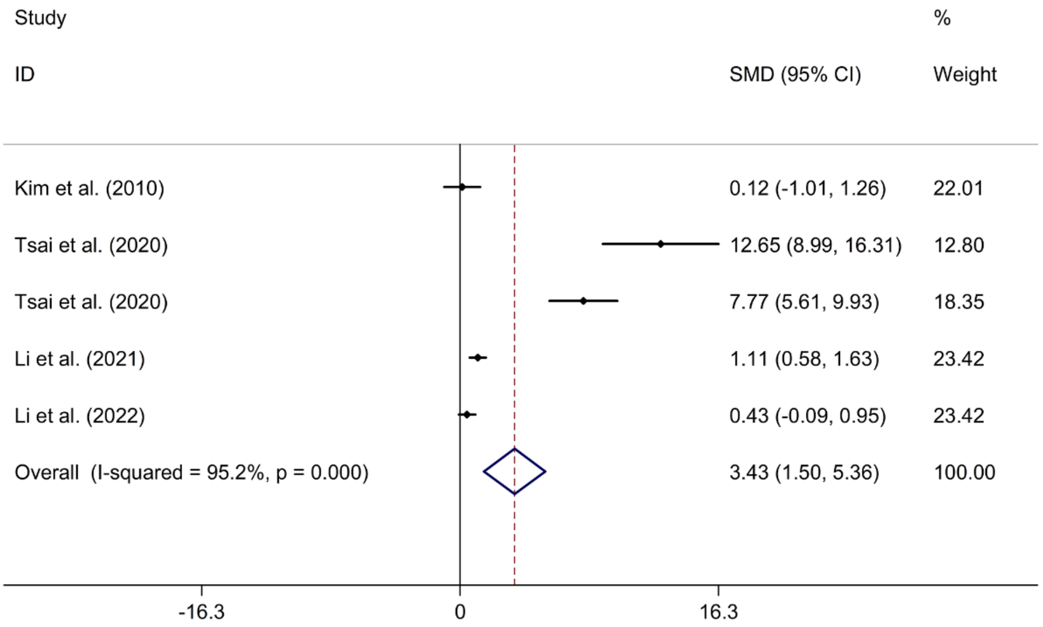

Supplementary Figure 8. Forest plot regarding the immediate effect of rTMS on language function in PSCI. Abbreviations: CI, confidence interval; PSCI, post-stroke cognitive impairment; rTMS, repetitive transcranial magnetic stimulation; SMD, standard mean difference.
