# Supplementary material for: Effect of non-invasive brain stimulation on post-stroke cognitive impairment: a meta-analysis
Source: Front Neurol. 2024 Oct 16;15:1424792. doi: 10.3389/fneur.2024.1424792 (PMC11521814; doi:10.3389/fneur.2024.1424792)
Supplement: Supplementary file 16 [file Table_1.DOCX]

Supplementary Table 1. Characteristics of included studies regarding the effect of rTMS on PSCI.

| Study | Publication year | Sample size | Age (Years) | Gender (M/F) | Stroke  Type (ischemic/hemorrhagic) | Stroke  Location (left/right/bilateral) | Stroke Onset (day) | Intervention | Site of  Stimulation | Intensity of  Stimulation | Duration of  Stimulation | Treatment  Period | Outcome  Measure | Adverse  Control Effect |
| --- | --- | --- | --- | --- | --- | --- | --- | --- | --- | --- | --- | --- | --- | --- |
| Du et al. ([31](#_ENREF_31)) | 2005 | 30/30 | 57.6 ± 10.8 | 56.7% | NR | 15/10/5  16/10/4 | NR | rTMS + CT vs. sham rTMS + CT | Bilateral DLPFC | 60%RMT | 100 pulses | 0.5Hz, 4weeks, 5days/week | General CF | No |
| Fregni et al. ([21](#_ENREF_21)) | 2006 | 10/5 | 57.70 ± 11.27 | 80% | NR | 8/2/0 | 105.6 ± 87.9 | rTMS + CT vs. sham rTMS + CT | M1 | 100% RMT | 1,200 pulses | 1Hz, 1week, 5days/week | General CF | Anxiety,  Tiredness  mild  headache |
| Kim et al. ([22](#_ENREF_22)) | 2010 | 6/6/6 | 68.3 ± 7.4/53.5 ± 16.9 | 33.3%/66.7% | 5/1  4/2 | NR | 404.4 ± 71.7/241.2 ± 42.5 | rTMS + CT vs. sham rTMS + CT | Left DLPFC | LF: 80% RMT  HF: 90% RMT | 900pulses/ 450 pulses | LF: 1Hz, 2 weeks, 5 days/week  HF: 10Hz, 2 weeks, 5 days/week | General CF, attention, EF and language | No |
| Lu et al. ([14](#_ENREF_14)) | 2015 | 19/21 | 42.5 ± 12.3 | 63.2% | 8/11 | 1/8/0 | 67 ± 30 | rTMS + CT vs. sham rTMS + CT | Right DLPFC | 100% RMT | 600 pulses | 1 Hz, 4weeks, 5days/week | General CF, memory | Dizziness |
| Liu et al. ([15](#_ENREF_15)) | 2017 | 18/18 | 65.33 ± 7.05 | 61.1% | 5/13 | NR | 253.2 ± 53.7 | rTMS + CT vs. sham rTMS + CT | Left DLPFC | 100% RMT | 700 pulses | 10Hz, 4weeks, 5days/week | General CF | No |
| Li et al. ([16](#_ENREF_16)) | 2020 | 15/15 | 65.5 ± 3.7 | 46.7% | NR | 5/10/0 | 22.7 ± 8.1 | rTMS + CT vs. sham rTMS + CT | Left DLPFC | 90% RMT | 2,000 pulses | 5Hz, 3weeks, 5days/week | General CF | Dizziness, headaches |
| Liu et al. ([34](#_ENREF_34)) | 2020 | 29/29 | 58.6 ± 6.2 | 34.5% | 20/9 | 11/18/0 | 261 ± 54 | rTMS + CT vs. sham rTMS + CT | Left DLPFC | 100% RMT | 700 pulses | 10Hz, 4weeks, 5days/week | General CF, attention | NR |
| Tsai et al. ([35](#_ENREF_35)) | 2020 | 11/15/15 | 57.5 ± 12.3/ 60.1 ± 14.1 | 81.8%/73.3% | 8/3  8/7 | 11/0/0  15/0/0 | 999 ± 792/564 ± 606 | rTMS + CT vs. iTBS+ CT vs. sham rTMS + CT | Left DLPFC | 90% RMT | 600 pulses | 5Hz, 2weeks, 5days/week; 50/5Hz, 2weeks, 5days/week | General CF, visuospatial function, language, attention, memory | No |
| Yin et al. ([36](#_ENREF_36)) | 2020 | 16/18 | 56.7 ± 12.9 | 87.5% | 11/5 | 4/6/6 | 52 ± 38.3 | rTMS + CT vs. sham rTMS + CT | Left DLPFC | 60% RMT | 2,000 pulses | 10Hz, 4weeks, 5days/week | General CF, attention, EF, memory | No |
| Li et al. ([32](#_ENREF_32)) | 2021 | 33/32 | 61.8 ± 5.5 | 36.3% | 11/22 | 15/18/0 | 28.6 ± 12.6 | rTMS + CT vs. sham rTMS + CT | Contralateral DLPFC | 90% RMT | 1,000 pulses | 1Hz, 4weeks, 5days/week | General CF, visuospatial function, memory, language, attention | No |
| Chu et al. ([24](#_ENREF_24)) | 2022 | 21/20 | 57.24 ± 14.03 | 85.7% | 13/8 | 12/9/0 | 120 ± 150 | iTBS + CT vs. CT | Left DLPFC | 70% RMT | 600 pulses | 50/5Hz, 6weeks, 5days/week | General CF, visuospatial, attention | No |
| Li et al. ([33](#_ENREF_33)) | 2022 | 28/30 | 69.5 ± 12.7 | 57.1% | 18/10 | 12/16/0 | 25 ± 9.19 | iTBS + CT vs. sham-iTBS + CT | Left DLPFC | 100% RMT | 600 pulses | 50/5Hz, 2weeks, 5days/week | General CF, language, visuospatial function, memory, EF | Sneezing |
| Yingli et al. ([37](#_ENREF_37)) | 2022 | 18/18 | 60.39 ± 10.87 | 72.2% | 10/8 | 17/1/0 | 58.11 ± 28.89 | rTMS + CT vs. sham rTMS + CT | DLPFC of the unaffected hemisphere | 80% RMT | 600 pulses | 1Hz, 8weeks, 5days/week | General CF | No |

Abbreviations: CT, conventional therapy; CF, cognitive function; DLPFC, dorsolateral prefrontal cortex; EF, executive function; F, female; iTBS, intermittent theta burst stimulation; M, male; NR, not reported; RMT, resting motor threshold; rTMS, repetitive transcranial magnetic stimulation.
