# Supplementary material for: Effect of non-invasive brain stimulation on post-stroke cognitive impairment: a meta-analysis
Source: Front Neurol. 2024 Oct 16;15:1424792. doi: 10.3389/fneur.2024.1424792 (PMC11521814; doi:10.3389/fneur.2024.1424792)
Supplement: Supplementary file 17 [file Table_2.DOCX]

Supplementary Table 2. Characteristics of included studies regarding the effect of tDCS on PSCI.

| Study | Publication year | Sample size | Age (Years) | Gender (M/F) | Stroke  Type (ischemic/hemorrhagic) | Stroke  Location (left/right/bilateral) | Stroke Onset (day) | Intervention | Site of  Stimulation | Intensity of  Stimulation | Duration of  Stimulation | Treatment  Period | Outcome  Measure | Adverse  Control Effect |
| --- | --- | --- | --- | --- | --- | --- | --- | --- | --- | --- | --- | --- | --- | --- |
| Park et al. ([19](#_ENREF_19)) | 2013 | 6/5 | 65.3±14.3 | 66.7% | 4/2 | 2/4/0 | 29.0±18.7 | anodal tDCS  + CT vs. sham tDCS  + CT | prefrontal | 2.0mA | 30 min/day | 5days/weeks, until the discharge since the hospitalization at the rehabilitation center | General CF, attention, EF | NR |
| Yun et al. ([29](#_ENREF_29)) | 2015 | 15/15/15 | 60.9 ± 12.9/58.9  ± 15.0 | 40%/46.7% | 7/8  9/6 | 6/6/3  5/6/4 | 42.2 ± 31.9/38.1  ± 27.0 | anodal tDCS of the left anterior temporal lobe + CT vs. anodal tDCS of the right anterior temporal lobe + CT vs. sham tDCS+ CT | Anode: left  fronto-temporal  area/anode: right  fronto-temporal  area | 2.0mA | 30 min/day | 5days/weeks, 3  weeks | General CF, attention, EF, memory | NR |
| Sun et al. ([28](#_ENREF_28)) | 2016 | 26/27 | 56±9 | 65.4% | 26/0 | NR | 37±16 | anodal tDCS  + CT vs. no tDCS + CT | prefrontal | 1.5mA | 20 min/ day | 6days/weeks, 4  weeks | General CF, attention | NR |
| Shaker et al. ([27](#_ENREF_27)) | 2018 | 20/20 | 54.45 ±  4.68 | NR | NR | NR | 421.5 ± 45.9 | tDCS  + CT vs. sham tDCS + CT | Anode: left or right  DLPFC cathode:  contralateral area | 2.0mA | 30 min/ day | 3days/weeks, 1  month | General CF, attention, memory | NR |
| Zeng et al. ([30](#_ENREF_30)) | 2019 | 15/15 | 56.21 ±  9.11 | 60% | NR | NR | 41.29 ±  10.37 | tDCS  + CT vs. sham tDCS + CT | Left DLPFC | 2.0mA | 20 min/ day | 5days/weeks, 4  weeks | General CF, visuospatial function, EF, attention | NR |
| Ai et al. ([20](#_ENREF_20)) | 2021 | 14/13/14 | 61.64 ±  10.33/61.36 ±  8.51 | 78.6%/76.9% | NR | NR | 54.25 ± 46.62/33.39 ± 15.33 | tDCS  + CT vs. sham tDCS + CT | Anode: left DLPFC  cathode: right  supraorbital area;  tDCS treatment and  conventional  rehabilitation at the  same time/separate  tDCS treatment and  conventional  rehabilitation | 2.0mA | 30 min/ day | 5days/weeks, 2  weeks | General CF, attention, EF | NR |
| Liu et al. ([25](#_ENREF_25)) | 2021 | 20/20 | 63.72 ±  8.41 | 60% | NR | NR | 75 ± 39 | tDCS  + CT vs. sham tDCS + CT | Anode: left DLPFC  cathode: right  DLPFC | 2.0mA | 20 min/ day | 5days/weeks, 4  weeks | General CF, attention, EF | NR |
| Liu et al. ([26](#_ENREF_26)) | 2021 | 25/25 | 65 | 60.0% | 10/15 | 16/9/0 | 240 | anodal tDCS  + CT vs. sham tDCS + CT | Anode: left DLPFC  cathode: right  DLPFC | 2.0mA | 20 min/ day | 5days/weeks, 4  weeks | General CF, attention, EF | No |
| Chu et al. ([24](#_ENREF_24)) | 2022 | 19/20 | 61.58 ± 14.18 | 73.7% | 14/5 | 12/7/0 | 60 | tDCS + CT vs. CT | Anode: left DLPFC  cathode: right  DLPFC | 2.0mA | 20 min/ day | 5days/weeks, 6  weeks | General CF, visuospatial function, attention | No |
| Ko et al. ([17](#_ENREF_17)) | 2022 | 12/14 | 61.25 ±  12.85) | 33.3% | NR | NR | NR | tDCS  + CT vs. sham tDCS + CT | Anode: left DLPFC  cathode: right  supraorbital area | 2.0mA | 30 min/ day | 5days/weeks, 4  weeks | General CF, attention, EF | NR |
| Zhang et al. ([18](#_ENREF_18)) | 2023 | 30/30 | 55.70 ± 6.22 | 53.3% | NR | NR | NR | tDCS  + CT vs. no tDCS + CT | DLPFC | 2.0mA | 20 min/ day | 5days/weeks, 4  weeks | General CF, attention, Language, EF, language, memory | Mild adverse reactions |

Abbreviations: CT, conventional therapy; CF, cognitive function; DLPFC, dorsolateral prefrontal cortex; EF, executive function; F, female; M, male; NR, not reported; tDCS, transcranial direct current stimulation.
